# IVC Filters in Integrated Acute Pulmonary Embolism Management—A Narrative Review

**DOI:** 10.3390/jcm14196810

**Published:** 2025-09-26

**Authors:** Joseph P. Hart, Mark G. Davies

**Affiliations:** 1 Center for Quality, Effectiveness, and Outcomes in Cardiovascular Diseases, Houston, TX 77054, USA; mark.davies@ascension.org; 2 Division of Vascular Surgery, Medical College of Wisconsin, 8701 Watertown Plank Rd., Milwaukee, WI 53226, USA; 3 Department of Vascular and Endovascular Surgery, Ascension Health, Waco, TX 76710, USA

**Keywords:** pulmonary embolism, IVC filters, management, outcomes

## Abstract

Acute pulmonary embolism (APE) remains a significant cause of mortality and morbidity despite increasing prophylaxis for deep venous thrombosis (DVT). The IVC filter is a temporary or permanent intravascular device that traps migrating thrombi from their origin in the pelvis or a lower limb into the pulmonary vasculature, thereby preventing significant APE. The current and longstanding indications for placing an IVC filter are in patients with documented lower extremity DVT and acute APE who also have absolute contraindications to anticoagulation or have experienced an acute, hemodynamically unstable APE requiring ventilatory and vasoactive support, with limited cardiovascular reserve. Updated guidelines have led to a significant rise in IVC filter placements for specific therapeutic indications of venous thromboembolism compared to prophylactic use. Meta-analyses show that IVC filter placement is associated with a lower risk of subsequent APE but an increased risk of DVT. However, there appears to be no significant reduction in APE-related mortality and no change in all-cause mortality. Early complications after IVC filter placement typically relate to procedural issues and include bleeding or infection at the venous access site, development of arteriovenous fistulas, accidental arterial puncture, and post-procedural access site hematoma or thrombosis. Additional early complications include IVC filter malposition, incomplete expansion, IVC penetration, or guidewire entrapment. Delayed complications may involve DVT below the filter, IVC occlusion due to the filter, IVC filter migration, fracture of one of the IVC filter components, IVC rupture, or IVC thrombosis. Retrieval of IVC filters by simple, advanced, or open techniques should be considered after weighing the risk-to-benefit for the individual patient. Deployment of the IVC filter remains an important component of interventional APE management within the narrow indications currently proposed. Current guidance recommends that an untethered temporary IVC filter should be placed and retrieved once the contraindication to anticoagulation is resolved.

## 1. Introduction

Acute pulmonary embolism (APE) continues to drive significant morbidity and mortality notwithstanding documented increasing utilization of prophylaxis for deep venous thrombosis (DVT) within inpatient services [1,2]. Appropriate clinical use of inferior vena cava (IVC) filters has proven invaluable, mitigating sequalae of APE in subjects documented to have DVT [3]. The primary role of the IVC filter is to interrupt central migration of a thrombus from its point of origin in a limb into the pulmonary vasculature, which can lead to a clinically significant APE [4]. However, in the era of increasing interventional therapy, newer pharmaceutical agents, newer reversal agents for anticoagulants, and a greater understanding of the timing of anticoagulation, coupled with the risks associated with long-term indwelling IVC filters and the influx of retrievable and bio-convertible filters, the use of IVC filters in the integrated management of APE is evolving [5]. This review seeks to examine the current role of IVC filters in integrated acute pulmonary embolism care.

## 2. Anatomy of the IVC

The IVC is the largest vein in humans and is formed by the right and left common iliac veins at the L5 lumbar vertebral level. It ascends through the retroperitoneum, anterior to the spine, on the right side of the aorta, pierces the central tendon of the diaphragm at the level of T8, and enters the right atrium. It has four segments: hepatic, prerenal, renal, and postrenal segments. It has a length of 22–25 cm and a diameter of 20–30 mm. The IVC receives tributaries that parallel branches of the abdominal aorta (4 sets of lumbar veins, two Inferior phrenic veins, multiple hepatic veins, two or more renal veins, and a right gonadal vein). Anomalies of the IVC and its tributaries have a reported prevalence in the range of 0.3% to 10.1% and include Hypoplasia (0.005% to 1%), Aplasia (0.005% to 1%), IVC Duplication (0.2% to 3%), Left sided IVC (0.2–0.5%), megacava (>28 mm or >30 mm; 1% to 3%) and IVC Webs (<0.01%)

## 3. Indications and Contraindications for IVC Filter

The current and longstanding indications to place an IVC filter are for patients experiencing a documented lower extremity DVT and a documented acute APE who also have absolute anticoagulation contraindications or in patients who have experienced an acute hemodynamically unstable APE requiring ventilatory and vasoactive support and have limited cardiovascular reserve [6,7,8,9,10,11]. Individual patient considerations moderate medical decision-making on IVC filter placement, particularly for patients with a concurrent acute cardiopulmonary condition, hemodynamically unstable APE, documented poor residual cardiopulmonary reserve, or an anticipated duration of contraindication to anticoagulation [12,13]. These subsets of patients may also benefit from a retrievable IVC filter placement in the short term, with the goal of removing the IVC filter early. Thus, one can consider placement of an IVC filter as a preventive measure in the setting of a DVT and APE and a protective measure in patients whose cardiovascular reserve cannot suffer additional insults. The indications, both relative and absolute, for an IVC filter are shown in Table 1.

## 4. Societal and National Guidelines

There is broad consensus internationally that IVC filters should be placed in patients suffering from a DVT or acute APE with an absolute contraindication to anticoagulation.

The European Society of Cardiology [6] has identified potential indications for the insertion of an IVC filter as the presence of a DVT or APE with absolute contraindication to anticoagulants (Class IIa; Level C) and recurring APE despite adequate anticoagulation (Class IIa; Level C);

The American College of Chest Physicians (ACCP) guidelines [11,15] states that the presence of an acute proximal DVT or APE experiencing contraindication to anticoagulation represents an indication for IVC filter placement (grade 1B). The routine deployment of IVC filters in VTE without anticoagulant contraindication is not recommended.

The American Heart Association [9,14] has identified adult patients with any acute proximal DVT or APE with contraindications to anticoagulants or active bleeding (Class I, level of evidence B) Adult patients with PE or DVT recurrence while on therapeutic anticoagulation should also be considered for an IVC Filter (Class IIa; level of evidence C and Class IIb, level of evidence C, respectively).

The American College of Radiology 2019 document [8] has suggested three categories of patients where an IVC filter is indicated: acute proximal deep vein thrombosis of the leg or APE with absolute contraindication to anticoagulation, significant anticoagulation complications, or failure of anticoagulation, where a retrievable IVC placement is considered “usually appropriate” while a permanent device “may be appropriate”.

The National Institute for Health and Care Excellence (NICE) in the United Kingdom [16] specifically identified two scenarios where IVC filter may be considered: with contraindications to anticoagulation and VTE recurrence in patients taking anticoagulants

The Society of Interventional Radiology (SIR) has supported IVC filter placement in a select subset of patients with acute APE receiving treatment with complex therapies, including thrombolysis, percutaneous thrombectomy, or embolectomy, where clinical adverse event risk due to APE or recurrent PE exceeds potential downsides of IVC filter deployment [14].

## 5. IVC Filter Designs

The basic design principles of IVC filters are to maximize the trapping of a migrating thrombus from a lower extremity DVT while concomitantly permitting physiologic venous blood return through the IVC. This was the foundation of the first filters described by Mobin-Uddin in 1967 using an open technique and Greenfield in 1973 using a percutaneous technique [17,18]. The designs of IVC filters may be described as untethered non-retrievable (permanent filters) or retrievable filters and tethered retrievable IVC filters (retrievable filters). Untethered non-retrievable filters cannot be retrieved percutaneously and remain in the IVC permanently, with retrieval usually obtained through an open approach or conversion to endo-trash by percutaneous intervention if required. Untethered non-retrievable filters are designed to be captured and retrieved early, but can be retrieved later, often with the adjunctive use of lead extraction lasers or endovascular forceps techniques [4]. Newer filters can be converted from a closed filtering model to an open non-filtering mode using secondary endovascular interventions. This ability to change the mode of IVC negates the need to recover the filter [19]. In more recent design iterations, bio-convertible filters have been described wherein the indwelling device can convert to an “open” non filtering configuration, due to a bioabsorbable key component at center of the device in the IVC lumen that when intact secures the filter legs in a filtering mode but when the component is degraded over the initial 6 month period after implantation the filter arms are unlocked and retract against the IVC wall become incorporated by endothelialization leaving the IVC unfiltered [20]. In the last decade, the concept of a tethered percutaneous retrievable filter has been tested for us on unstable critically ill patients [21]. The design of this filter incorporated an IVC filter mounted on a triple-lumen femoral central venous line, allowing for insertion and retrieval in concert with the femoral line [22,23,24]. In the experimental arena, focus has turned to fully bioabsorbable filters, which will dissolve over time in a triggered or untriggered manner [25,26]. A further concept has been to introduce drug coating to the legs of filters to significantly delay bio-integration with the IVC wall and thus ease percutaneous retrieval of retrievable filters [27]. Animal studies of drug-eluting retrievable vena cava filters have shown promise. A new drug-elution retrievable IVC filter with a heparin-modified poly (ε-caprolactone) (hPCL) coating also containing rapamycin was applied by electrospraying. The in vitro drug release pattern demonstrated coating release over one month. in vivo results demonstrated that the drug-eluting retrievable vena cava filter provided anti-intimal hyperplasia properties at the filter wall interface, which significantly improved the retrieval rate and allowed for an extended in vivo dwell time within sheep [28]. A second innovative hydrogel coating including 10 mg/mL heparin and 30 mg/mL cyclodextrin/paclitaxel (PTX) inclusion complex (IC) was utilized, enabling sustained release over approximately two weeks. In vivo, the drug-coated retrievable vena cava filter had a markedly better retrieval rate at 67% compared to bare retrievable IVC filters (0%) at 4 weeks. [29].

## 6. IVC Filters and the Spectrum of Venous Thromboembolism (VTE)

Liu et al. [30] in a recent systematic review evaluated the majority of IVC filter studies as having a low risk of bias using the Cochrane Collaboration tool. Performance bias was considered a high risk in all the studies. For the primary outcome of mortality, the outcome assessment was unlikely to be biased by the presence or absence of blinding, and we estimated the detection bias as low risk. However, the National Institute for Health and Care Excellence in the United Kingdom [16] stresses the lack of reliable studies and that the vast majority of the studies are graded as low- or very low-quality

In the PREPIC study, 400 patients who were diagnosed with a proximal DVT and considered to harbor elevated risk for pulmonary embolization (PE) underwent randomization to placement of a permanent IVC filter as well as anticoagulation or to anticoagulation alone [31]. A reduction in the occurrence of pulmonary embolism compared with anticoagulation only. However, this beneficial effect was counterbalanced by a significant increase in deep-vein thrombosis; in addition, the presence of an IVC filters had no impact on mortality, compared with those treated with anticoagulation alone. A second randomized controlled study, the PREPIC 2 [32], recruited 399 patients with APE and symptomatic high-risk DVT who received anticoagulation therapy for at least 6 months. Patients were randomized to receive or not receive a retrievable IVC filter, with a planned removal at 3 months. The findings showed that IVC filter placement in patients who could receive anticoagulation was not beneficial. At 8 years, the presence of a vena cava filter reduced the risk of pulmonary embolism but increased that of deep-vein thrombosis and had no effect on survival. The success rate of IVC filters in preventing APE from lower extremity DVT is reported to be around 98% [33]. In subjects with recurrent VTE while on anticoagulant therapy [34], the placement of an IVC filter reduced all-cause mortality and mortality due to pulmonary embolism at 3 months in patients who suffered recurrent APE. Patients with uncomplicated recurrent DVT did not benefit from placement of an IVC filter.

The success rate of IVC filters in preventing APE from lower extremity DVT is reported to be around 98% [33]. In subjects with recurrent VTE while on anticoagulation treatment [34], the placement of an IVC filter reduced all-cause mortality and mortality due to pulmonary embolism at 3 months in patients who suffered recurrent APE. Patients with uncomplicated recurrent DVT did not benefit from placement of an IVC filter. There are several observational studies [35,36] that have examined the role of IVC filter placement in patients with poor cardiovascular disease who experience a VTE event. These reports identified an important reduction of in-hospital death rates (7.6% compared to 18%; filter vs. no filter) for patients with a hemodynamically unstable APE who received intravenous thrombolytic therapy and inferior vena cava filter placement. A second study by Stein et al. [37] showed that subjects having hemodynamically unstable APE treated with thrombolytic therapy or embolectomy, all-cause mortality was lower in patients who received an IVC filter compared to those who did not IVC filter (21% vs. 48%; filter vs. no filter). In contrast, Liang et al. identified no significant alteration in mortality in hemodynamically abnormal patients with APE given systemic thrombolytics with or without IVC filter placement [38]. In Predicting the Safety and Effectiveness of Inferior Vena Cava Filters (PRESERVE), a prospective, nonrandomized study (1421 participants) with 24 months of follow-up, implantation of IVC filters was associated with few adverse events and with a low incidence of clinically significant APE [39]. Contraindications to anticoagulation therapy or failure of anticoagulation were the indication in 81.6% while 8.9% IVC filters were considered prophylactic. IVC filters were removed successfully in 44.5% of the patients. After IVC filter removal, non-fatal venous thromboembolic events occurred in 6.5% patients (DVT—5.2%, APE—1.6% and/or IVC thrombotic occlusions—1.1%). The results of the PRESERVE study demonstrate that IVC filters are safe and effective in preventing clinically significant APE.

## 7. Short-Term and Long-Term Complications of IVC Filters

While complications of IVC filters may occur in the immediate post-insertion window, they more commonly arise after the first month, and their risk increases over time [40]. Complications can be considered either short-term and long-term (Table 2).

Short-term complications are related to the procedure complications and include bleeding or infection at the venous access site, occurrence of femoral or other access s AV fistulas, inadvertent arterial injury or puncture, and peri-procedural access site hematoma or vessel occlusion. Additional adverse occurrences in the early period include IVC filter malposition, partial expansion, IVC penetration, or entrapment of a guidewire. Delayed events include DVT below the filter, IVC occlusion due to the IVC filter, IVC filter migration, fracture of one of the IVC filter components, IVC rupture, or IVC thrombosis. The rates of complications are shown in Table 3 and can be very variable with large ranges of reported rates.

*Access related events:* Complications due to vascular access for inferior vena cava filter insertion occur in 4–11% of cases [44]. Bleeding at the access site is seen in 6–15% of patients [45]. The occurrence of venous thrombosis at the site of insertion for an IVC filter access site has been reported to range from 2–35%. Venous thrombosis happens more often in subjects with an identified disorder of hypercoagulability [46]. However, few patients (3%) report symptoms [47]. The incidence of arteriovenous fistula (AVF) is a very unusual sequela of IVC filter placement (0.02%) and is thought to result from inadvertent entry into adjacent arteries (carotid or femoral arteries) during the initial placement procedure. The observed incidence rate, reported in a review of published case series through 2004, was 0.02% [48].

*IVC Filter Tilt:* During or following IVC filter placement, the IVC filter can tilt in the IVC and IVC filter tilt is defined as greater than 15 degrees angulation of the filter from the long axis of the IVC. Tilt has been reported in 0.7% to 13.9% cases [45]. With an IVC filter tilt of <15 degrees, there is not an elevated risk of thrombosis. In IVC filter tilt of >15 degrees there is a trend toward increased APE and IVC thrombosis [44,49] IVC filter tilt ≥5.0 degrees, dwell time and age are associated with challenging retrieval [50].

*IVC Filter migration:* Important filter movement is considered as a 2 cm or more cephalad or caudal displacement from the initial/intended placement site. There are a variety of causes of IVC filter migration, including under-sizing, failure to adhere to the wall, and disturbance from intravascular wires and catheters. With modern IVCFs, the incidence of migration is markedly reduced, with all filters demonstrating <1% migration incidence, exclusive of the G2 filter, which has shown a 4.5% migration incidence. It was noted that 90% of episodes of migration were identified over one month after initial placement [3].

*Incomplete opening of the filter:* Incomplete IVC filter opening is most likely related to a defect in the filter, deployment error, or an unidentified IVC thrombus. This results in an asymmetric and/or abnormal filter configuration within the IVC. This phenomenon is reported in 0.7% to 13.9% of cases [50]. Incomplete opening of the IVC filter is associated with an 80% reduction in filtering efficacy, particularly for <5 mm thrombi due to gaps in the filtering configuration [51].

*IVC Thrombosis:* Thrombosis of the IVC has been cited to occur in under 10% of cases using contemporary filters [46,52]. Analysis of the problem across various filter designs demonstrates no significant difference in rates of IVC thrombosis below [45,53,54]. Importantly, the placement of retrievable filters has a higher incidence of IVC filters compared to permanent filters [55]. The presence of malignancy does not affect IVC thrombosis secondary to an IVC Filter [56]. Once IVC thrombosis is discovered, it is common practice to start anticoagulation if appropriate; however, no important difference in rates of regression or progression of the thrombus has been reported [57]. Prophylactic anticoagulation does not better the incidence of IVC thrombosis [48]. IVC thrombosis can be treated by thrombolysis or thrombectomy of the filter thrombus and can be reduced to endo-trash by stenting across the filter and increasing flow through the IVC [58].

*Fracture of the IVC Filter*: Filter fractures are defined as a structural disruption of a filter with fragmentation and potential embolization of the fragment(s). Fracture of the filter is a late complication of an indwelling IVC filter and is seen most commonly after a filter has been indwelling for over 12 months [59,60,61,62]. The incidence is reported to be 1–2% [53,54,55,56,57,58,59,60,61,62,63]. Fragments that transit through the right heart present similarly to pulmonary embolism.

Intravascular filter fragments can be removed safely, with snares and forceps, with success rates that vary according to location [64]. Due to the fact that extravascular fragments are not readily accessible for removal, many patients cannot be rendered fragment-free [65].

*Filter Perforation:* This entity occurs when part of a filter spans more than 3 mm through the IVC wall and moves into the peri-caval space or adjacent structures [66,67,68,69]. IVC Filter-related IVC perforation accounts for about 20% of IVC filter-reported complications [42,70]. Of note, retrievable filters have increased rates of perforation compared to permanent filters, and in cases of retrievable filters, perforation depends on the IVC filter’s dwell time [55]. Use of anticoagulation in subjects with perforation of the IVC by filter component(s) increases bleeding risk and may drive retroperitoneal hematoma occurrence [71].

*Recurrence of DVT*: The occurrence of a DVT is recognized as a common delayed complication of IVC filter insertion. Multiple reports have shown that the chance of DVT varies with the type of filter and ranges from 4% to 18%, increasing with IVC filter dwell time. In the PREPIC study, the incidence of recurrent DVT in patients who received an IVC filter compared to those who did not was 20.8% versus 11.6%, respectively [31]. This difference between the two groups persisted out to 8 years of follow-up 2 [72]. A retrospective report encompassing more than 80,000 patients admitted for DVT demonstrated that subjects undergoing caval filter insertion suffered higher incidence of recurrent DVT at 12 months compared to those not receiving caval filters (5.3% vs. 3.7%) [73].

*IVC filter occlusion*: Reports on incidence of IVC filter occlusion have suggested that the rate ranges from 6% and 30% [74]. A recent meta-analysis has suggested that the introduction of new-generation filters has been associated with a lower incidence of filter thrombosis (2.8%) [3]. Independent predictors of IVC filter occlusion have been reported as new or propagated DVT at follow-up, no antiplatelet therapy at follow-up, internal jugular venous access, the presence of VTE on the index admission, and temporary IVCF placement.

## 8. Current Outcomes of IVC Filter Placement

Bikdeli et al. performed meta-analysis of six randomized controlled trials (RCTs) and 5 prospective controlled observational studies comparing use of IVC filters to no intervention in subjects at risk for APE in 2017. The quality of evidence for the examined RCTs spanned low to moderate. After placement of an IVC filter, patients had a decreased chance of subsequent APE (OR: 0.50), but an elevated chance of DVT (OR: 1.70). There was no significant reduction in APE-related mortality (OR: 0.51), and no change in overall mortality (OR: 0.91) [75]. In an updated review and meta-analysis by Liu in 2021, six randomized controlled trials with 1274 total patients (638 IVC filter patients and 636 control subjects) met their criteria from the inception of databases to 31 October 2019. There was no significant difference in mortality due to APE between the IVC filter and control groups by 3 months or during all of follow-up, with low heterogeneity among the studies. The new occurrence of APE within 3 months and during the entire follow-up period was lower in the IVC filter group than in the control group (0.81% vs. 5.98%; *p* = 0.01; and 3.2% vs. 7.79%; *p* = 0.001, respectively). No significant differences were found in the rates of new DVT, major bleeding, or mortality during the full follow-up period between the two groups [30].

## 9. Current Usage

In the current era of retrievable devices, the placement of IVC filters has fallen over time. Within the United States (US), the overall incidence of IVC filter placement increased from 2002 to 2010, with a downward trend observed after 2010 [76]. The reason for the reduction in IVC filter usage in the US was the 2010 US Food and Drug Administration (FDA) safety communication, which was renewed in 2014. Those announcements were coupled with additional mandates on reporting IVC Filter-related adverse events [77]. In a study to assess modern trends in IVC filter utilization in the National Inpatient Sample (NIS) between 2005 and 2019, Yoo et al. [78] demonstrated an uptick in the IVC filter use from 2005 to 2010 which was reversed to a decreasing trend following the FDA communication on IVC Filters in 2010. This trend persisted following the second FDA communication in 2014. However, over the entire time examined, the proportion of IVC filters deployed for those having documented VTE rose sharply from 70.8% to 82.2%. In a further analysis of the Nationwide Inpatient Sample, the number of filter deployments related to a defined therapeutic VTE indication compared to prophylactic use increased significantly during the study period from 69.8% in 2005 to 80.4% in 2014 [79]. In the SAFE-IVC study of Medicare outpatient and inpatient claims data spanning 1 January 2013, to 31 December 2021, 270,866 patients were identified as having IVC filters placed: 65% were placed for an initial VTE event, 26% were placed for recurrent VTE, and 9% to effect VTE prophylaxis. Of note, these patients, 63% of these patients had a recorded major bleed or trauma within 30 days of IVC filter placement. Similarly to other studies, there was a decrease in the volume of IVC, which decreased from 44,680 per year in 2013 to 19,501 per year in 2021. The rate of IVC Filter retrieval was 15.3% at a median of 1.2 years and 16.8% at ultimate follow-up at 9.0 years. Factors that mitigated against IVC Filter retrieval were older age, increasing presence of comorbidities, and black race, whereas index device placement at an academic medical center resulted in an improved chance of IVC filter explant. The majority (94%) of removal attempts were successful, yielding a low 30-day complication rate (mortality, 0.7%); filter-related complications, 1.4% [80].

## 10. IVC Filter Retrieval

IVC filter removal should be considered in all IVC filter cases, regardless of their duration or apparent difficulty. IVC filter leg penetration is common; however, devices with the hook, apex, or collar embedded in or through the IVC wall are predictors of retrieval failure [81]. There are multiple techniques for retrieving IVC filters, ranging from simple to advanced methods (Table 4). Indications for using complex retrieval techniques include failure of standard retrieval and tilting or embedding in the IVC wall [82]. The more advanced retrieval methods are associated with higher failure rates compared to simpler techniques that involve snaring the hook of the IVC filter. A recent meta-analysis showed a combined success rate of 92.0% with retrievable filters and 96.4% with permanent filters. Three percent of patients experienced a major complication, with no association between the occurrence of major complications and the filter type [82]. In asymptomatic subjects having failed percutaneous IVC filter removal a low device-related complication rate in midterm follow-up is observed. Absent symptoms or other complicating events, these patients are not mandated to undergo open surgical filter removal. In contrast, symptomatic patients should be considered for open removal, with expected low morbidity and symptom resolution following operative filter explant [83].

## 11. Post IVC Filter Removal

Limited information exists about the IVC after filter removal. A classification system has been established to describe the IVC appearance on venocavogram following filter retrieval, based on luminal characteristics and the presence of extravasation. Luminal narrowing is graded from 1 to 4: Grade 1—0% to 50%; Grade 2—50% to 99%; Grade 3—occlusion; and Grade 4—avulsion. Extravasation is classified as Grade A—no extravasation; Grade B—contained extravasation; and Grade C—free extravasation. A study by Li et al. demonstrated that after IVC filter removal, 97.3% had Luminal narrowing grade 1, 1.3% grade 2, 1.3% grade 3, and none had grade 4. No extravasation was observed in 96.4% (grade A); however, 2.7% had extravasation grade B, and 0.9% grade C [84].

## 12. Conclusions

**Figure 1:**
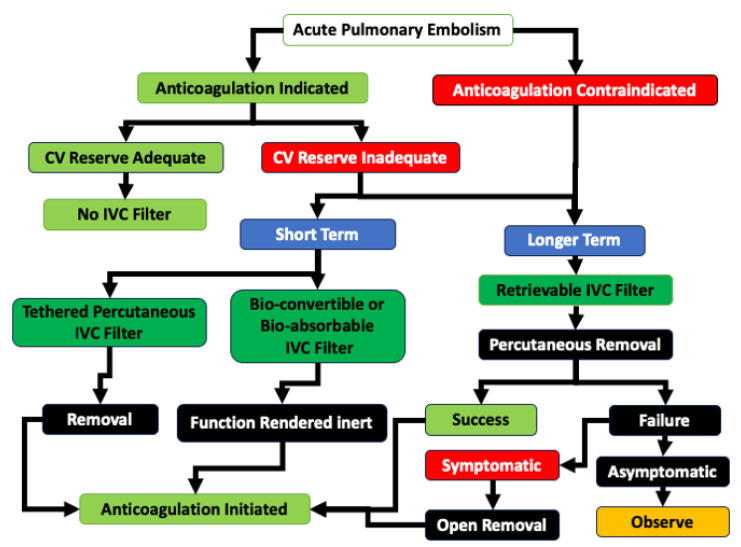
Algorithm for management of IVC filters in Acute Pulmonary Embolism. In the absence of contraindications, anticoagulation is indicated. If the patient has inadequate cardiovascular (CV) reserve, a short-term filter (a tethered or a bio-absorbable/bio-convertible filter) can be considered to provide protection against a subsequent APE. If there are contraindications to anticoagulation, placement of a retrievable IVC filter is recommended, and earlier percutaneous removal is standard of care. If there is failure of standard retrieval techniques, advanced percutaneous techniques or open removal can be considered. The impetus for removal is dependent on symptoms, current APE risk, and risk-to-benefit considerations of the removal procedure. This algorithm is a synthesis of the current literature.

There has been a shift in the use of IVC filters in the current era, with a greater emphasis on patients with documented lower extremity DVT and APE who also have absolute contraindications to anticoagulation or have experienced an acute, hemodynamically unstable APE requiring ventilatory and vasoactive support, with limited cardiovascular reserve. While deployment of the IVC filter remains an important component of interventional APE management, IVC filter placement is now constrained within the narrow indications currently in effect, and should be performed within a structured and dedicated system to retrieve IVC filters in order to reduce complications. Current guidance recommends that an untethered temporary IVC filter should be placed and retrieved once the contraindication to anticoagulation is resolved. A proposed algorithm for integrated management of IVC filters in Acute Pulmonary Embolism in shown in Figure 1.

## Figures and Tables

**Table 1:** Absolute and Relative Indications for IVC Filter Placement [6,9,11,14,15].

Absolute	Relative
Acute VTE and absolute contraindication to anticoagulation Anticoagulation failure IVC Aplasia and Hypoplasia	VTE with Poor Cardiopulmonary Reserve Adjunct to thrombolysis in patients with Sub-massive/Massive APE Prophylaxis in high-risk patients So called “Free-floating” Caval or Iliac Thrombus Iliocaval DVT planning to undergo thrombolysis Severe uncorrectable coagulopathy Bacteremia Megacava: >28 mm

**Table 2:** Short-term and Long-term Complications of IVC Placement.

Short-Term	Long-Term
Bleeding Access site thrombosis Arteriovenous fistula IVC Filter Tilt Incomplete opening of IVC Filter Malpositioning of the IVC Filter Embolization of the IVC Filter	Recurrent DVT IVC Filter migration IVC Thrombosis IVC Filter Fracturing IVC Filter Perforation IVC Filter Thrombosis

**Table 3:** Rates of Complications for IVC Filter Placement [41,42,43].

Complication	Reported Rates
Insertion site Complication Access site Thrombosis IVC Filter Malpositioning IVC Filter Migration IVC Filter Fracture IVC Penetration IVC Perforation IVC Thrombosis Recurrent APE	5–23% 1–25% 1.0–9.0% 1–18% 2–10% 1–41% 1–20% 1–10% 0.5–6.0%

**Table 4:** Short-term and Long-term Complications of IVC Filters.

Retrieval Technique	Indication
Regular retrieval with snare	IVC filters with a free hook in uncomplicated position.
“Wire under filter” technique	IVC filters lacking a hook or with a hook lodged in the IVC wall.
Multiple sheaths technique	long indwelling IVC filters resistant to the standard techniques to bolster the retrieval unit and prevent buckling of the sheath
Laser Catheter	Long dwell time IVC filters resistant to the standard techniques to release the struts from the IVC wall.
Endobronchial forceps	Long indwelling IVC filters resistant to the standard techniques to release the struts from the IVC wall
Compression to Endo-trash	Long indwelling IVC filters resistant to the standard and advanced techniques to compress the filter against the IVC wall
Open Surgical Removal	Long indwelling IVC filters with perforation, fragmentation, or resistant to the standard and advanced techniques

## Abbreviations

APE	acute pulmonary embolism
IVC	Inferior Vena Cava
IVCF	Inferior Vena Caval Filter
ECMO	extra-corporal membrane oxygenation
DVT	deep venous thrombosis
VTE	venous thromboembolism
